# Virtual microscopy enhances the reliability and validity in histopathology curriculum: Practical guidelines

**DOI:** 10.15694/mep.2019.000028.1

**Published:** 2019-02-13

**Authors:** Samal Nauhria, Lee Hangfu

**Affiliations:** 1 Windsor University School of Medicine

**Keywords:** Computer-based Assessment, Digital Pathology, Learning outcomes, Undergraduate education, e-learning/computers, Teaching & Learning

## Abstract

This article was migrated. The article was not marked as recommended.

Digital pathology innovation and application in medical education have paved the path for a significant shift in the advancement of the medical curriculum. The new technology of virtual microscopy is a proven reliable and valid pedagogy method for histopathology learning objectives, and assessments. The current transformation has brought educators around the globe nearer towards the goal of achieving competence in Curriculum Inventory in the medical curriculum. This paper emphasises the practical tips and guidelines for cost-effective implementation and the successful use of Virtual Microscope technology to enhance the histopathology curriculum in a medical school.

## Introduction

Virtual Microscopy (VM) is one facet that has revolutionised learning histopathology worldwide. Whole Slide Imaging (WSI) technique makes use of modern slide scanners and Virtual Microscopy (VM) software. The process involves digitisation of glass slides to a high-resolution format which can be conveniently viewed using specialised VM software on a computer or handheld tablet devices. VM software reproduces a high-quality image with meticulous clarity and added features that allow students to highlight, annotate, and pan and zoom (up to a maximum of x100) WSI. ( Banavar et al. 2016)

VM in undergraduate histopathology education promotes various integrated active learning and discussion activities during small‑group laboratory sessions. It has also been adopted in the field of cytology, haematology, continuing medical education (CME) and delivering of research journal content. ( Dee 2009) There is ample research data to prove that the use of VM has enhanced student learning and overall performance in a more clinically oriented and dynamic learning environment ( Triola and Holloway 2011; Pantanowitz et al. 2012; Brierley et al. 2017; Vainer et al. 2017; Dominick et al. 2018). This proven beneficial learning technology has been highly accepted and adopted by several medical schools across the globe. The United States Food and Drug Administration (USFDA) recently approved the use of VM for diagnostic purposes ( Boyce 2017). Thus, VM can also provide a modality that helps bridge the gap for students with inadequate clinical exposure.

The accreditation governing committees is advancing curriculum inventory (mapping) in the medical school to standardise the educational objectives of the required medical curriculum ( Russ et al. 2013; Knollmann-Ritschel et al. 2017). Several Medical Programs around the world have adopted the VM to compliment the competency‑based education (CBE) in their medical education (Lurie 2011; Gruppen et al. 2012; Jolly et al. 2013; Russ et al. 2013). Digital pathology has paved a path to address some of these core competencies and collaborative models in various medical education programs ( Triola and Holloway 2011; Saco et al. 2016). However, the utilisation of VM is entirely new to some medical school pedagogy at the basic science level.

Recently, the USFDA has authorised the marketing of the Philips IntelliSite Pathology Solution (PIPS), which is the first WSI system for interpreting digital surgical pathology slides from biopsy tissue samples ( Boyce 2017). As evident, with the increasing use of VM for histopathology diagnosis, it is imperative that students become proficient in the system applications.

The author would like to present the practical tips on how this transition can be successfully achieved for a medical school. Table 1 presents an outline of these tips and impact of implementation at each step.

**Table 1.** Implementing virtual microscopy for a basic sciences pathology course

Practical Implementation fact	Impact of strategy
Consider technology requirements and initial setup	Efficient set up of in-school and online IT infrastructure is essential for seamless access to the slide boxes and VM software. Cost-efficient methods include access to free online slides sources
Acquisition of slides: Ensure quality and credibility	Selection slides that depict microscopic features from common disease processes
Faculty development for technology use	Training faculty to operate the VM software ensures superior delivery of this instructional technology
Address student bias towards this new technology	Currently, VM is being used for diagnosis so adapting to this newer technology during early medical school years will benefit students in their medical career
Orientation to slide box and software	Discussion on “how to navigate WSI” ensures students understand how to use technology to enhance medical knowledge
Slide selection for a session: Align with course objectives	Careful slide selection make histopathology learning more meaningful for the students
Slide annotations allow for Standardization, Reliability and Validity	Personalized learning material enhances comparable and collaborative learning activities for both faculty and students
Clinical case-based group discussions enhance knowledge and communication	Clinical vignettes attached to a slide provoke self-centred and team-based learning
Online take home MCQ based quizzes enhance formative assessment	Trigger further interest in students and increased use of personal mobile devices create an “anytime and anywhere” platform
Avoid cognitive overloading: limit slides per session	Cognitive overload can lead to a decline in information assimilation by the human brain. Limiting to 4-5 slides per session can thus limit cognitive overload
Storage of data and backup	Safe and secure data servers ensure smooth access to the slides while preventing any loss of data
Collect student feedback and surveys	Using feedback from students to enhance curriculum is an essential component of “continuous quality improvement” for all medical schools

## Practical Tips and Guidelines

### Tip 1

#### Consider technology requirements and initial setup

The high cost of slide scanners is still a significant disadvantage for adopting this system. However, a medical school can very quickly acquire high-quality slide boxes for a meagre price. The easy availability of the online resources which are cloud-based can also be used for teaching purposes at the basic sciences level. The pathology department at our University acquired the IOWA online slide box which is an inexpensive option available for teaching use. The Aperio ImageScope used as the VM software is available as a free download from the internet (Aperio ImageScope - Pathology Slide Viewing Software: Leica Biosystems 2019)

These slides are set up on a network drive attached to the server. The local server is linked to computer systems in the histopathology lab by virtual computing (N-computing) which is again a cost-effective option available for use. A primary server is sufficient to handle a load of around 30-40 students at one time which can be upgraded as required. A cloud-based server is a better alternative however, efficiency varies with the internet speeds. A minimum connection speed of 10mbps is sufficient to handle cloud-based WSI. The cloud-based server is configured for remote access allowing the student to access these slides at a time and place of their choice in addition to the histopathology lab. Few advantages of using VM are outlined in Table 2.

**Table 2.** Potential advantages of using virtual microscopy vs light microscopy

Application	VM	LM
**Ease of use**	Easy navigation of slides by a VM software	Higher skill level is required for operating a light microscope
	Possibility of individual and collaborative annotations	Potentially spoils the glass slides if markers are used to highlight areas of interest
	High-Resolution slide Images	Normal to low resolution with minimal quality control
**Accessibility and storage**	Can be stored and shared online	The only possibility is to share photos of slides without quality assurance
	Digital media can be stored forever in a local or cloud server without fear of loss of slides	Fragile glass slides are often lost or broken
**Implications for student and faculty**	Student interest higher	Outdated and boring method
	Ample free online/cloud-basedresources	Limited resources available in non-interactive format
	Lesser faculty load in VM based instruction in medical schools	More faculty needed for LM based teaching
**Impact on healthcare**	Better Quality control	Cannot ensure quality
	Worldwide collaboration possible	Restricted to sharing of slides or slide pictures

### Tip 2

#### Acquisition of slides: Ensure quality and credibility

Make sure that the acquired set of WSI is a high-quality slide set, which contains slides that depict microscopic features from common cellular changes seen during a disease process. The IOWA Virtual Slide Box contains around 1000 slide images for teaching both histology and pathology in basic sciences courses. In the modern Internet era, medical informatics around the globe has facilitated rapid communication among medical students and their ability to discuss and share strategies across institutions, regions and countries. The development of VM and its adaptation in medical education has accommodated to the need for standardisation in tissue sections by maximising comparability, reliability, repeatability and validity in learning objectives ( Dee et al. 2003; Kumar et al. 2004; Pinder et al. 2008; Farah and Maybury 2009; Husmann et al 2009; Weaker and Herbert 2009; Fónyad et al. 2010; Barisoni et al. 2017).

### Tip 3

#### Faculty development for technology use

The lab instructors need to be trained initially to operate the computer system and navigate slides through the VM software. The current Aperio ImageScope VM software used at our university comes with a cost-free option for viewing the slide images. The software allows the students to adjust magnification, pan and zoom, annotate, perform image analysis, and compare different stains. A minimal effort of time with a basic level computer system and software skill is sufficient to operate VM software. Additional demonstration and training sessions can be requested from the software vendor. A reasonable motivational factor for teaching faculty is that lesser effort and time is required to deliver VM based interactive sessions ( Dee 2009; Foster 2010; Pantanowitz et al. 2012; Tian et al. 2014; Fonseca et al. 2015; Sagol et al. 2015; Saco et al. 2016).

### Tip 4

#### Address student bias towards this new technology

Student bias must be addressed immediately and effectively against the use of WSI and VM software. After the USFDA approval of this technology for diagnosis in 2017, the students are bound to encounter this system sometime in their medical career. The early introduction of the VM software can provide a modality that will bridge the gap of inadequate clinical exposure during the career of a student.

Moreover, the WSI and VM system allows physicians from anywhere in the world to collaborate and consult with each other confirming a diagnosis. Thus, early exposure to VM will prepare students to work efficiently with advanced patient health information systems, in which they will be able to view histopathology images and assist in the appropriate diagnosis of a patient.

### Tip 5

#### Orientation to the slide box and software

The next important step is to familiarise the student to the VM software and the WSI slide box. The faculty should demonstrate the use of this technology in real time on a bigger screen or projector connected to a computer. Ensure that all students can access slides and the related clinical information associated with a slide. It is vital to prepare students for what they are about to see in the slide box and software. Questions or discussions before exposure to this new methodology will achieve the necessary knowledge for students to process the learning objective from each slide ( O’Neill and Wyness 2005).

### Tip 6

#### Slide selection for a session: Align with course objectives

The course director can select and identify the slide which depicts the microscopic features aligned to the specific learning objectives discussed in a didactic lecture. These can further be mapped to the institutional and medical education objectives. Brierley et al. (2017) have discussed VM use for enhancing the medical curriculum in a CBE system by the successful integration of pathology into clinical scenarios. Eventually elevating student learning and making histopathology more consequential to students.

Kumar et al. (2006) have used this teaching methodology for vertically integrating histology and pathology courses by providing access to normal histology slides along with slides depicting disease processes. This creates an opportunity to improve the understanding in the relationship between morphological changes and the clinical manifestations of a disease.

### Tip 7

#### Slide annotations allow for Standardization, Reliability and Validity

VM technology has allowed medical schools to establish digital laboratories, which allow students and teachers to build unique and personalised learning materials, i.e. annotations ( Pinder et al. 2008; Husmann et al 2009; Fónyad et al. 2010; Triola and Holloway 2011; Helle et al. 2013; Tian et al. 2014).

All advanced VM software allow individual student annotations on the slides images, which can be saved for further discussion and can also be reviewed by the faculty for assessments. On the other hand, annotating the glass slide would require as many glass slides as the number of students repeatedly. Above all, there is no possibility of standardisation as different slides depict different features and a student may lose out on the opportunity to view and identify the same features. The possibility of annotations by VM software provides an avenue to annotate the slide, discussion of the microscopic features, assessments and future use as these annotations can be deleted from the software.

Annotations facilitate the implementation of comparable and collaborative learning activities involving both students and faculty. The students can share annotations on a common image using any device, from any location achieving the need for standardisation, reliability and validity in medical pathology education ( Helle et al. 2013; Sahota et al. 2016). Comparable deployment of annotation activities in the medical curriculum will positively improve the student understanding of microscopic morphology in pathology pedagogy ( Sahota et al. 2016).

### Tip 8

#### Clinical case-based group discussions enhance knowledge and communication

At our university, a clinical case-based learning vignette is provided along with the supporting slides. The associated clinical history, lab values and radiological findings along with other relevant clinical information are assimilated at different levels of discussion, thus provoking self-centred and team-based learning. This is followed by group discussions on the case and students are free to look up for information from any resource of their choice. This active learning process provides a chance for the student to develop competencies in communication skills and group dynamics using problem-based learning pedagogy that is so vital in the clinicopathological matrix of integrated learning ( Janssen et al. 2009; Brierley et al. 2017)

### Tip 9

#### Online take home MCQ based quizzes enhance formative assessment

Lab assessments based on Multiple Choice Questions (MCQ’s) can be used to trigger further interest in the students. At our University, we use questions from an existing question bank comparable to the NBME questions. The assessment consists of MCQ’s and short‑answer type questions based on the microscopic description, differential diagnosis, or clinical features of the disease. This activity thus measures different aspects of learning which includes medical knowledge, combined comprehension and application, and problem‑solving ability of the students.

Making these MCQ based assessments accessible and available online provide an opportunity for self-learning, cognitive acquisition and clinical competency in the student. Hartman (2015) has described the use of a smartphone, and personal mobile devices are popular digital pathology platforms to decrease contact hours without eliminating content while maintaining effective pedagogical methods ( Thompson and Lowrie 2017). Several universities who have adopted this methodology have shown a reduction of time spent in conducting VM based laboratory sessions ( Triola and Holloway 2011; Gatumu et al. 2014).

### Tip 10

#### Avoid cognitive overloading: limit slides per session

Ensure a limited number of available slides per session. During each interactive session, students at our university discuss and review information for 4-5 histopathology slides in a two-hour session. The student is required to describe the clinical or radiographic image, suggest a differential diagnosis, describe the histopathological image and attempt to determine a final diagnosis with a treatment plan.

Verbal and visual channels in our brain are processed separately thus limiting the amount of simultaneous information assimilation. Mayer and Moreno (2003) have discussed a detrimental impact on memory if the information presented exceeds this capacity. Integrating visual and verbal channels avert cognitive overloading ( Martin 2014) and thus have important implications for the optimal design of instructional technology.

The structure of the VM session should facilitate students’ ability to organise content into a coherent cognitive structure, to integrate it with relevant prior knowledge, and to apply the information in new situations to solve problems.

### Tip 11

#### Storage of data and backup

It is advisable to store the entire data on the firewalled server with updated antivirus software. A properly configured firewall will restrict access to everything except the specific services that need to remain open. Backing-up and protecting the server with a secure and reliable storage solution is essential to ensure that data is not lost.

At our University, the Instructional Technology Committee (ITC) serves the Information Technology (IT) needs of both faculty and students. The ITC department regularly communicates with the concerned faculty and students for smooth operation.

### Tip 12

#### Collect student feedback and surveys

Collecting student feedback is an important aspect of successful implementation of this technology for teaching use in a medical school. Various studies have concluded with positive feedback from students regarding the use of VM ( Goldberg and Dintzis 2007; Triola and Holloway 2011; Cogdell et al. 2012; Tian et al. 2014; Bridge et al. 2015; Leifer 2015; Van Es et al. 2015).

Wayne et al. (2013) report that regardless of prior academic ability, students who reported a positive perception of their school’s learning environment performed better on a standardized exam than did students who reported less positive perceptions.

At our university, student feedback is collected at the end of the semester using google forms. The questionnaire is based on modified statements adapted from the six ACGME competency criteria that a student should acquire during his/her medical career. (Appendix) After the student feedback, some of the original histopathology cases have also been amended thus leading to a student-centred instructional approach.

## Summary

With the evolution of digital pathology, this technology will undoubtedly serve as a valuable tool for improving the standardisation, reliability, and validity in the histopathology pedagogy. There are numerous documented advantages of using this new technology assisting physicians in the medical diagnosis of clinical-pathology diseases. The benefits of VM are undoubtedly essential for medical students, pathologists, lab technicians and researchers to be proficient in using this new technology.

## Take Home Messages

• This article is focussed primarily on the easy implementation process of virtual microscopy for teaching purpose in undergraduate and postgraduate medical schools.
• VM use has proven to be a practical solution and has the potential to transform the process of both teaching and learning histopathology.
• With the increasing use of VM for pathology diagnosis, it is imperative that students become proficient in using these systems.

## Notes On Contributors

Dr. Samal Nauhria, M.D. Pathology, is the Chair of Pathophysiology department at Windsor University School of Medicine, Saint Kitts. His research is focussed on using computer-assisted instruction and implementation of newer applications to enhance or replace traditional teaching strategies and address many new pragmatic and pedagogical challenges in medical education. ORCID: https://orcid.org/0000-0001-7373-2606

Dr. Lee Hangfu, M.D., is the Dean of Clinical Sciences at Windsor University School of Medicine, Saint Kitts. In addition to teaching clinical medicine, his research is focussed on student-based integrated medical pedagogy. ORCID: https://orcid.org/0000-0001-7626-6268
