# Supplementary material for: Virtual microscopy enhances the reliability and validity in histopathology curriculum: Practical guidelines
Source: MedEdPublish (2016). 2019 Jun 19;8:28. Originally published 2019 Feb 13. [Version 2] doi: 10.15694/mep.2019.000028.2 (PMC10712629; doi:10.15694/mep.2019.000028.2)
Supplement: Peer review history

# Reviewer report: Reviewer response for version 2

**DOI:** 10.21956/mep.20242.r31388

**Published:** 2020-03-26

**Authors:** Manish Khazane

**Affiliations:** 1 SRM UNIVERSITY,RAMAPURAM,CHENNAI.

This review has been migrated. The reviewer awarded 4 stars out of 5 The authors have aptly putforth their views in the present article about the aid by technology in medical education. In the coming months, we can believe that medical education and the whole aim of equipping medical personals for the future can be achieved more prominently.The spread of the COVID-19 has brought a stark reality to the human kind that change is constant. And this should spring us back in action with better ways of teaching, learning and treating our patients. The ubiquitous availability of video and audio accessories in all our technologies including laptops, mobile phones has eased this difficult phase.The use of VM as a aid to teach seems one of the promising ways in the present scenario. Achieving our aim of continuity in medical learning as well as safeguarding our resources ( in the form of health care workers of the future ) against any harm.

Reviewer Expertise:

NA

No decision status is available

# Reviewer report: Reviewer response for version 2

**DOI:** 10.21956/mep.20242.r31386

**Published:** 2019-10-15

**Authors:** Trevor Gibbs

**Affiliations:** 1 AMEE

This review has been migrated. The reviewer awarded 3 stars out of 5 I would agree with my co-reviewers and reflect upon my original review, which considers this paper to be an interesting and important paper for those faculty tasked to teach histology. Its teaching elements appear innovative and effective, with the paper written in a clear fashion.I do however still worry that such a paper needs to expand on how the teaching of histology ( an important subject) can and should be integrated into the modern curricula which has the danger of being over-crowded with often unused scientific knowledge.

Reviewer Expertise:

NA

No decision status is available

# Reviewer report: Reviewer response for version 2

**DOI:** 10.21956/mep.20242.r31387

**Published:** 2019-06-24

**Authors:** Daniel Brierley

**Affiliations:** 1 University of Sheffield

This review has been migrated. The reviewer awarded 4 stars out of 5 I enjoyed reading this paper which summarised some of the key areas which require attention before implecement VM into curricula. There are certainly many areas to consider if the technology is to be used to its maximum potential. The ability to acquire virtual teaching sets is especially beneficial to those centres that don't have access to large databases of previously scanned material.

Reviewer Expertise:

NA

No decision status is available

# Reviewer report: Reviewer response for version 2

**DOI:** 10.21956/mep.20242.r31385

**Published:** 2019-06-20

**Authors:** Ken Masters

**Affiliations:** 1 Sultan Qaboos University

This review has been migrated. The reviewer awarded 5 stars out of 5 A much-improved paper on the topic. I am pleased to see that the authors have addressed the issues raised about Version 1. Table 1 might seem a little strange, and possibly would be better delivered as an end glossary, but it does serve the very useful purpose of explaining the technical terms. Those readers who are unfamiliar with the terminology can refer to it, whereas others can gloss over it.Table 2 is also a fine contribution to the paper, enhancing the points made later and providing a strong literature basis. (It is a pity that there is some information missing from the listing (Saco et al., 2016), but that is minor issue.) With these as background context (and some of the language changes made in the main text), the rest of the paper immediately becomes far more accessible and valuable to the reader.

Reviewer Expertise:

NA

No decision status is available

# Reviewer report: Reviewer response for version 1

**DOI:** 10.21956/mep.18991.r27227

**Published:** 2019-02-18

**Authors:** Trevor Gibbs

**Affiliations:** 1 AMEE

This review has been migrated. The reviewer awarded 3 stars out of 5 There is no doubt that this paper is of value to those considering setting up an histology programme in a health Sciences programme. I would support my co-author in his comments about the technology description and the need for both clarity of defining the terms and the use of English.However my worry with this paper and its relationship to modern approaches to medical education is how it fits into our approach to relevance if subjects within a crowded undergraduate programme and how it integrates into future learning needs. Are we not / should we not be moving away from such silo approaches to learning. Of course this approach probably counts more within a postgraduate programme that is specifically designed more for the postgraduate pathologist.

Reviewer Expertise:

NA

No decision status is available

# Reviewer report: Reviewer response for version 1

**DOI:** 10.21956/mep.18991.r27228

**Published:** 2019-02-14

**Authors:** Amrinder Singh

**Affiliations:** 1 Millcreek Medical Clinic

This review has been migrated. The reviewer awarded 5 stars out of 5 This paper is complete and thorough. Very nicely written. I highly recommend this article.

Reviewer Expertise:

NA

No decision status is available

# Reviewer report: Reviewer response for version 1

**DOI:** 10.21956/mep.18991.r27229

**Published:** 2019-02-14

**Authors:** Ashwani Naaz

**Affiliations:** 1 Health department pb. Govt.

This review has been migrated. The reviewer awarded 5 stars out of 5 This artical is too good and precise . I recommend all connected with the field to read .

Reviewer Expertise:

NA

No decision status is available

# Reviewer report: Reviewer response for version 1

**DOI:** 10.21956/mep.18991.r27230

**Published:** 2019-02-14

**Authors:** sudesh naaz

**Affiliations:** 1 pb.govt. Health department

This review has been migrated. The reviewer awarded 5 stars out of 5 This article is very beneficial to the medical students.I strongly recommend this for all medical students to read this article.

Reviewer Expertise:

NA

No decision status is available

# Reviewer report: Reviewer response for version 1

**DOI:** 10.21956/mep.18991.r27226

**Published:** 2019-02-13

**Authors:** Ken Masters

**Affiliations:** 1 Sultan Qaboos University

This review has been migrated. The reviewer awarded 3 stars out of 5 The paper is a valuable contribution to guiding faculty in the use of this crucial technology. Because of the nature of the topic, the paper is necessarily technical in nature, and is also reasonably well-grounded in the technical and educational literature.Two areas of weakness, however, would need to be addressed in a revised version of this paper:• Technical references: I have already referred to the fact that the paper is necessarily technical in nature. The authors need to remember, however, that the average reader of the journal would have little experience with sophisticated computing terminology. Concepts like virtual and cloud-based computing are not standard terms in medical education, and many educators would have their eyes glaze over at some of the discussion. These terms need to be explained, carefully, and clearly. (In this vein, in the line “by virtual computing (N-computing)”, it would be unclear whether the “N” is an error, or whether it is a brand or technical term (referencing thin-client NComputing)). The authors need to clarify this, especially considering the audience most likely to be readers of the journal. Similarly, a line that speaks of “A minimum connection speed of 10mbps” is mostly meaningless to the average reader. The authors are advised to compare this speed to something with which the reader may be familiar, or give an indication of how long a 10 Mb file would take to download at this speed, and perhaps give an indication of a cost. Without points of comparison, these are just numbers. I would recommend that the authors have a non-technical colleague read through the paper, and identify all areas that are problematic.• The paper also suffers from a large number of small, but irritating, errors of language, punctuation and expression. I would strongly recommend that authors have the paper closely proof-read by an English mother-tongue speaker to remove these errors.Although I feel that the content of the paper is valuable, I am awarding three stars only. I feel that, once the authors have had a chance to address these issues (and issues raised by other reviewers) in a revised paper, then a higher star-rating may be awarded.

Reviewer Expertise:

NA

No decision status is available
